# Combining Calcium Hydroxylapatite and Hyaluronic Acid Fillers for Aesthetic Indications: Efficacy of an Innovative Hybrid Filler

**DOI:** 10.1007/s00266-021-02479-x

**Published:** 2021-08-02

**Authors:** Nabil Fakih-Gomez, Jonathan Kadouch

**Affiliations:** 1 Department of Facial Plastic & Cranio-Maxillo-Facial Surgery, Fakih Hospital, Khaizaran, Lebanon; 2 Practice for Aesthetic Dermatology, ReSculpt Clinic, Amsterdam, The Netherlands

**Keywords:** Hyaluronic acid, Calcium hydroxylapatite, Radiesse, Fillers, Hybrid, Premixing, Effectiveness

## Abstract

**Background:**

Limited data are available describing effectiveness of combining the use of calcium hydroxylapatite (CaHA) and hyaluronic acid (HA).

**Methods:**

The authors performed a retrospective chart review of patients injected with a premixed combination of CaHA and a cohesive polydensified matrix (CPM^®^) HA (CaHA:CPM-HA ) in the authors’ aesthetic practices. The midface and lower face were injected. Patients’ records were evaluated, and treatment results were scored using the Merz Aesthetics Scale for the jawline^®^ (clinician rated, CR-MASJ). Adverse events were recorded.

**Results:**

A total of 41 patients were included, all females with a mean age of 47.5 years (range 21–63 years). The mean CR-MASJ score improved from 2.12 at baseline to 0.68 at *t* = 3 months (SD = 0.69, 95% CI 1.28–1.60) and 1.27 at* t* = 12 months (SD = 0.74, 95% CI 0.43–0.74). 100% of the subjects had experienced a ≥1-point improvement in CR-MASJ score at t = 3 months, versus 85% at* t* = 12 months. No adverse events were reported.

**Conclusion:**

The results of this study support the volumizing and lifting potential of the hybrid mix CaHA:CPM-HA for treatment of cheeks and jawline.

**Level of Evidence IV:**

This journal requires that authors assign a level of evidence to each article. For a full description of these Evidence-Based Medicine ratings, please refer to the Table of Contents or the online Instructions to Authors www.springer.com/00266.

## Introduction

Hyaluronic acid-based injectable fillers (HAs) are currently the golden standard for volumization procedures in facial rejuvenation. Calcium hydroxylapatite (CaHA [Radiesse^®^]; Merz Pharmaceuticals GmbH, Frankfurt, Germany) is the second most used facial filler [1, 2]. The effect of HAs is mainly based on strategic deposition of filler in the different facial tissue layers resulting in volumization. Due to its compressible nature, HAs are considered ideal some physicians to use in areas where bone structures are (still) well-defined or skin is thin [3]. The effect of CaHA is mainly mediated by neocollagenesis, inducing indirect volumization, tissue-lifting and skin-tightening [2, 4–6]. Both types of fillers are generally believed to have excellent safety profiles [2, 7].

**Fig. 1:**
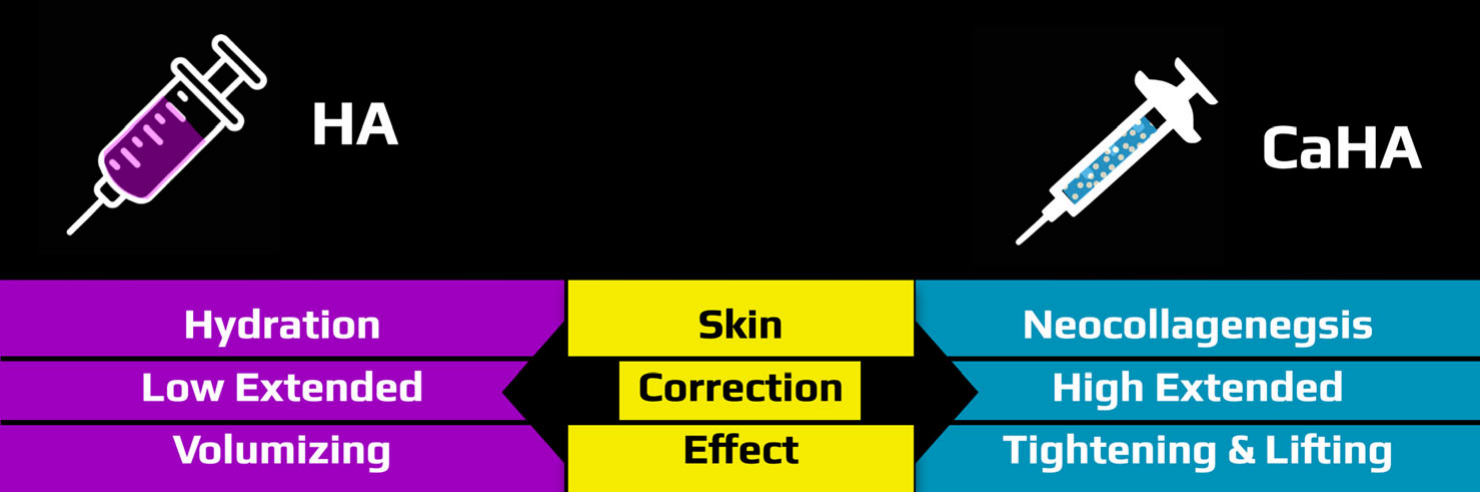
Many physicians are taking advantage of the complementary mechanisms of action of HA and CaHA fillers by combining

Many medicines or medical devices, in both elective as non-elective fields of medicine, are used for off-label indications [8]. The same accounts for injectables. Many physicians are taking advantage of the complementary mechanisms of action of HA and CaHA fillers by combining them, so that subjects receive both fillers in a single injection session (Fig. 1) [3, 9–13]. This can be achieved by layering the products in the same treatment area, or injection of a premixed combination of the two products. The main difference is that in the first option both products keep their distinct rheological properties, whereas in the second the rheological properties are changed in a way that has not been defined yet.

Due to its neocollagenesis properties, CaHA has a stronger tissue-lifting and skin-tightening effect compared to HAs. And because of its rheologic properties, such as a high G’ and high viscosity, it is known to help define bony definitions [3, 14]. Premixing HA with CaHA can add the neocollagenesis properties to a HA filler, whereas a high G prime HA can enhance a CaHA filler by adding additional volumization while securing tissue softness. In addition, CaHA-treated areas can sometimes undergo unexpected early volume loss due to rapid absorption of the carboxymethylcellulose gel carrier before the CaHA particle-induced neocollagenesis has taken effect [11]. When premixing HA with CaHA, the HA can compensate for this early volume loss. On the other hand, CaHA is known to have a longer effect than most Has [4, 11]. On the long-term, we hypothesize that premixing CaHA with HA can prolong the effect of the filler treatment.

Only limited studies have described the safety and efficacy of such treatments [3, 9–11, 13]. In a previous study, the authors reviewed published data on the safety of using CaHA and HA combinations for aesthetic indications and performed a safety-analysis in a retrospective chart review of 134 patients injected with a premixed combination of CaHA and a cohesive polydensified matrix (CPM^®^) HA in the authors’ aesthetic practices [13]. In the current study, the authors investigate the change for improvement and the result duration of the premixed CaHA:CPM-HA combination. No validated photo-numerical grading scale exists for the assessment of a lifting effect. Available grading scales evaluate the result of facial volume loss and tissue laxity/ptosis, such as the depth of the nasolabial fold or marionet lines, or the volume excess at the jowl [15, 16]. Since deformities along the jawline, such as volume and contour loss at the mandibular angle, jowling and the appearance of the prejowl sulcus, can be considered the endpoint of facial aging-induced volume loss and soft tissue ptosis [16–18], the authors chose to assess the jawline contour in order to score the volumization and lifting effect of the premixed CaHA:CPM-HA combination.

## Methods

### Study Design and Primary Endpoint

This study was designed as a multicenter retrospective cohort study, using chart reviews of patients that came seeking a lifting effect without the need to undergo surgery or threads. Treatment exclusion criteria were <18 years of age, pregnancy or intent for pregnancy, breastfeeding, any inflammatory of infectious (bacterial, viral, or fungal) condition of the face, suspected allergy for components of the used fillers or lidocaine, known auto-inflammatory or autoimmune diseases and treatment with botulinum toxin or fillers in the past 12 months. In addition, the availability of pre- and post-treatment (*t* = 3 months and *t* = 12 months) patient pictures, taken by the clinic staff, was required for inclusion. All study patients provided written informed consent before treatment. The study was conducted in accordance with guidelines of the Declaration of Helsinki (1996) and good clinical practice.

**Fig. 2:**
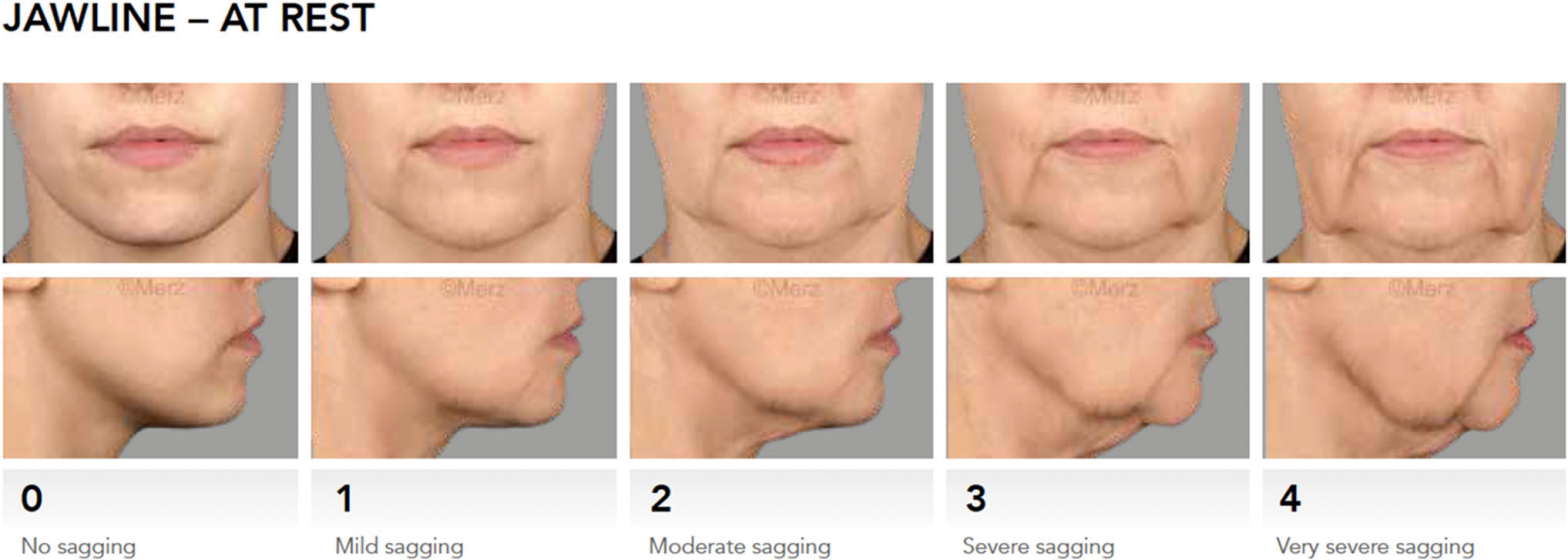
Merz Aesthetics Scale® for jawline: a validated, objective, quantitative rating scale for evaluating the esthetic signs of aging

Documented data included age, sex, health issues, treatment indication, used ratio for premixing, injection sites, injection technique, injection depth, injected volume, and the occurrence of adverse events. The primary endpoint was a 1-point improvement in baseline scores for jawline contour quantified by the validated photo-numeric 5-point Merz Aesthetics Scale^®^ for jawline as assessed by the injected physician (CR-MASJ, Fig. 2). The CR-MASJ was assessed pre-treatment, at *t* = 3 months and *t* = 12 months. Subjects with *a* ≥1-point improvement in CR-MASJ score were classified as treatment responders.

### Intervention

The used premixing protocol and treatment strategy was identical to the one described by authors in their previous publication on this topic [13]. The authors used a premixed hybrid formulation of CaHA and a hyaluronic acid composed of a cohesive polydensified matrix (CPM^®^, Belotero^®^ Volume, Merz Pharmaceutical GmbH, Frankfurt, Germany): CPM-HA Volume (CPM-HA V). The two products were mixed by placing the contents of a CaHA syringe and a CPM-HA V syringe using a Luer-lock connector into a 10-ml empty syringe. For every 1.5 cc CaHA, 0.5 cc of lidocaine 2% was added. A further 10-cc empty syringe was then joined, and the two gels transferred from one syringe to another, at least 10 times, to ensure full homogeneity. The ratio of the two products depended on the treatment indication and severity at presentation. The ratios of CaHA:CPM-HA in the face could vary from 1:1 to 1:3 for slight correction, from 1:4 to 2:4 for mild correction, and from 2:6 to 3:8 for severe correction (the numbers refer to the number of syringes, not the volume). The midface and lower face were injected. Only subcutaneous injections (layer 2 according to Mendelsons layering terminology) [19] using a fanning technique (0.1–0.2cc per trace) with a 25G x 50 mm canula were performed.

### Adverse Events

In the event of adverse events, they were to be classified according to the US FDA definition as immediate, early, or delayed. Immediate adverse events are those that present in the order of minutes to hours post-procedure. Early adverse events are those presenting within days to weeks. Delayed adverse events are defined as those presenting months to years later.

## Statistical Analysis

The assessed data are retrospective of nature. For this reason, the patients’ jawline contour images (using the Merz Aesthetics Scale^®^ for jawline) were all rated twice, once by the treating physician and once by an independent physician. An appropriate index that reflects both degree of correlation and agreement between ratings is the intraclass correlation coefficient (ICC). In this study, the index was calculated based on a two-way random effects model [REF]. Further analysis of the acquired CR-MASJ scored, patient characteristics and injected volumes was performed using RM ANOVA and a logistic regression analysis (Chi-square test).

## Results

A total of 41 patients could be included, all females with a mean age of 47.5 years (range 21-63 years). Injected CaHA:CPM-HA volumes ranged from 3.5 to 15.0 cc (mean = 9.61, median = 9, SD = 2.94).

### Interrater Reliability of the CR-MASJ Assessments

**Fig. 3:**
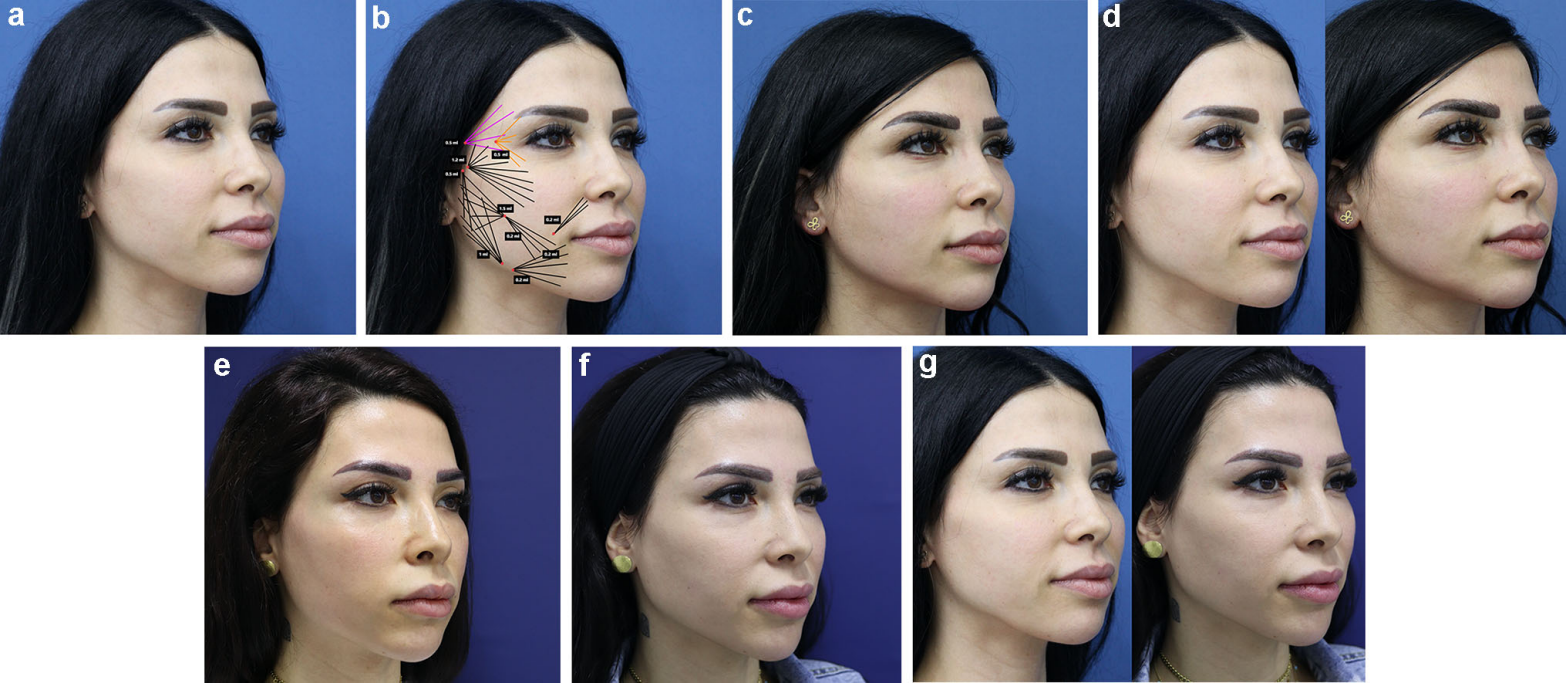
**a** 37-year-old female seeking full facial rejuvenation. **b** Premixed hybrid filler injection (3 cc of CaHA, 6 cc of CPM-HA V and 1 cc of lidocaine 2%) (black) is injected subcutaneously with threading technique. Also 0.5 cc of CPM-HA V (purple) was injected to the temples in each side and 0.5 cc of CPM-HA B (orange) in lateral orbital rim and tear valley on each side. **c** Result at 1 month after premixed hybrid filler injection. **d** Before and after 1 month after premixed hybrid filler injection. **e** Result at 6 months after premixed hybrid filler injection. **f** Result at 12 months after premixed hybrid filler injection. **g** Before and after 12 months after premixed hybrid filler injection

**Fig. 4:**
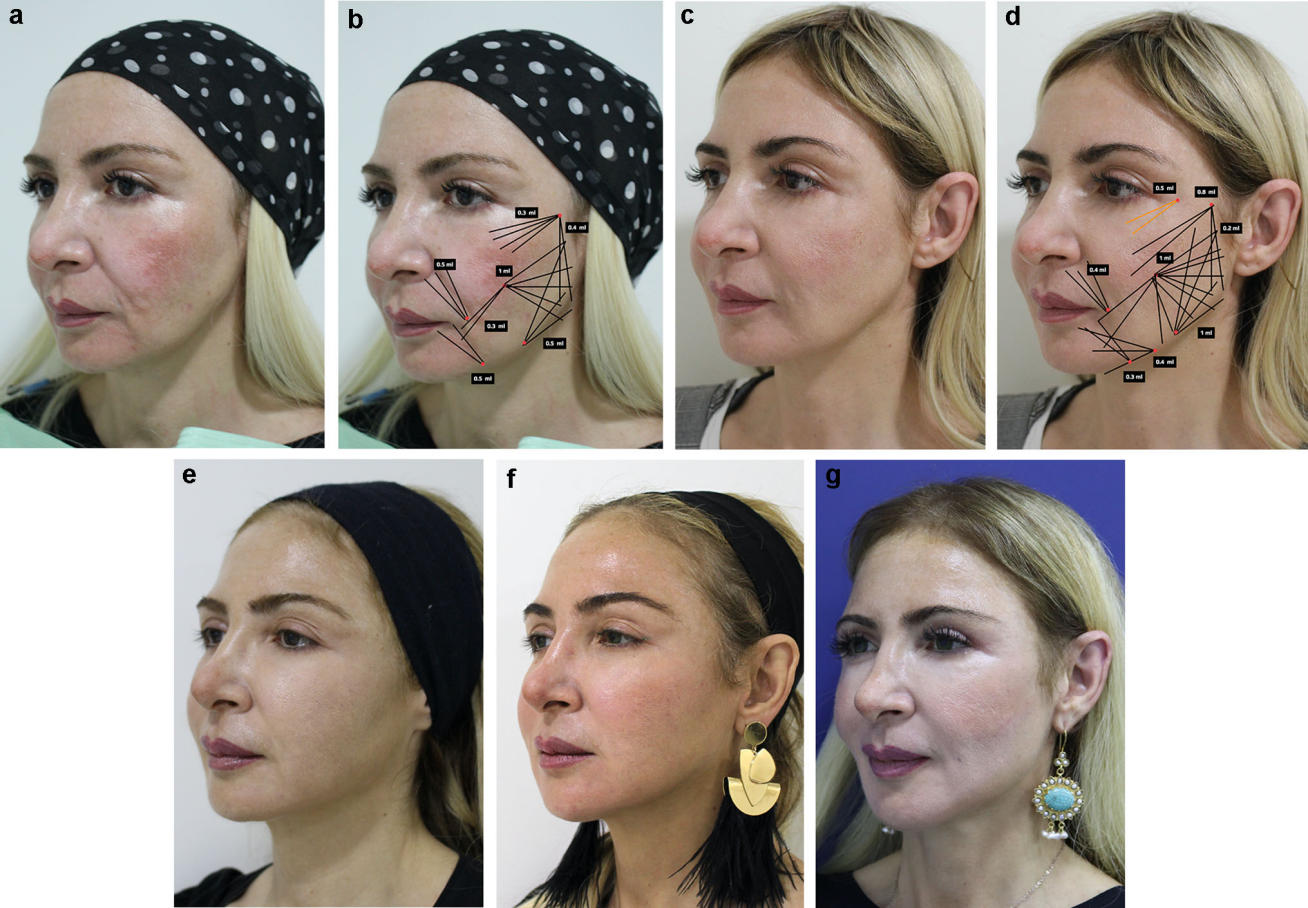
**a** 46-year-old female seeking full facial rejuvenation. **b** Premixed hybrid filler injection (3 cc of CaHA, 3 cc of CPM-HA V and 1 cc of lidocaine 2%) (black) is injected subcutaneously with threading technique. **c** Result at 1 month after premixed hybrid filler (3 cc of CaHA, 3 cc of CPM-HA V and 1 cc of lidocaine 2%). **d** Revision at 1 month after previous premixed hybrid filler injection 3 cc of CaHA, 3 cc of CPM-HA V and 1 cc of lidocaine 2%. Patient desired more improvement. Re-injection of premixed hybrid filler (3 cc of CaHA, 4 cc of CPM-HA V and 1 cc of lidocaine 2%) (black) and 0.5 cc of CPM-HA Balance (orange) in tear valley per side. **e** Result at 6 months after premixed hybrid filler injection (6 cc of CaHA, 7 cc of CPM-HA V and 2 cc of lidocaine 2%) and 0.5 cc of CPM-HA balance in tear valley per side. **f** Result at 12 months after premixed hybrid filler injection. **g** Result at 14 months after premixed hybrid filler injection

The patients’ jawline contours were assessed at baseline, at *t* = 3 months and *t* = 12 months (Figs. 3, 4). To assess the agreement between the assessed CR-MASJ score of the treating physician and once by an independent physician, interrater reliability analysis was performed using the intraclass correlation coefficient (ICC). The index was calculated based on a two-way random effects model [20, 21]. Agreement between the two raters was good to excellent; the ICCs of the CR-MASJ ratings at baseline and post-treatment (*t* = 3 months and *t* = 12 months) were 1.00, .92, and .90, respectively. Since the agreement was high, we only used the CR-MASJ scores based on the ratings of treating physician for analyses.

### Improvement in Facial Contouring of CaHA:CPM-HA Treatment

Baseline CR-MASJ scores were assessed pre-treatment, with a mean score of 2.12 (SD = 0.81, range 1–4). At baseline, CR-MASJ scores correlated, as would be expected, substantially with patient’s age (*r* = 0.64) and injected filler volume (*r* = 0.63), reflecting that older patients have stronger deformities along the jawline and stronger deformities are associated with larger filler injection volumes. After treatment with CaHA:CPM-HA, there were two post-treatment CR-MASJ measurements, one at *t* = 3 months and one at *t* = 12 months.

### Primary Endpoint

To assess the change of the mean CR-MASJ score over the two post-treatment measurements, a repeated-measures analysis of variance (RM ANOVA) was conducted. Although the CR-MASJ scores over the three measurement occasions and also the residual scores of the statistical model appeared reasonably normally distributed, with comparable (co)variances across measurements, we nevertheless also performed the nonparametric Friedman test to see if it supported the RM ANOVA results.

**Fig. 5:**
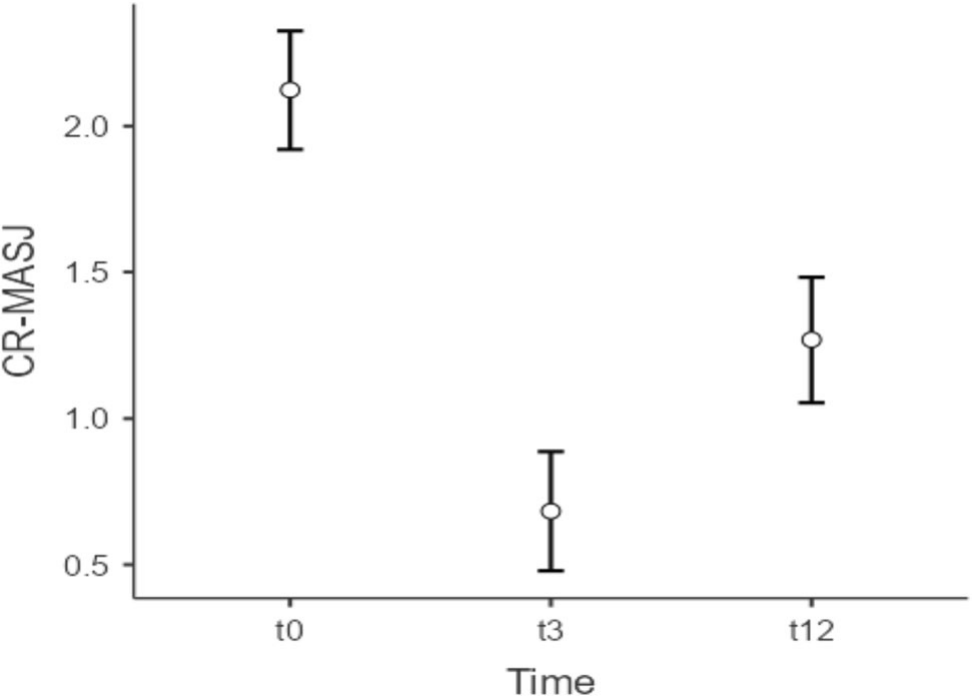
Mean CR-MASJ scores (with 95% confidence intervals) across baseline and post-treatment (*t* = 3 months and *t* = 12 months) measurements

At the first follow-up at *t* = 3 months, the mean CR-MASJ score had gone down by 1.44 points (95% CI 1.28–1.60) to a mean of 0.68 (SD = 0.69). With a change of almost two standard deviations, this may be judged as a considerable improvement. In the next follow-up, at *t* = 12 months, the CR-MASJ score increased relative to *t* = 3 months by 0.59 points (95% CI 0.43–0.74) to a mean of 1.27 (SD = 0.74). This relapse in the CR-MASJ score was noticeable, although it was still 0.85 points (95% CI 0.74–0.97) below the baseline level. The RM ANOVA showed that the differences in mean CR-MASJ scores across the pre-test and two post-test measurements were statistically significant, *F*(2, 80) = 204.73, *p* < 0.001. This was also found when using the Friedman test (Chi-square = 71.79, df = 2, *p* < 0.001). Following the RM ANOVA, (Bonferroni corrected) pairwise comparisons indicated that all three differences in CR-MASJ were statistically significant (all *p*-values < .001) (Fig. 5).

**Fig. 6:**
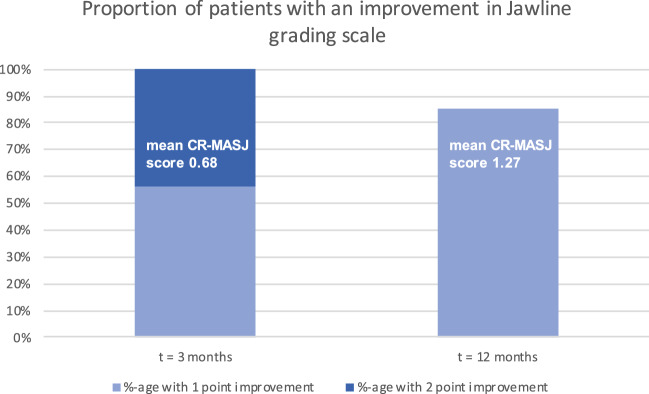
Improvement in jawline contour quantified by the validated photo-numeric 5-point Merz Aesthetics Scale for jawline^®^ (clinician rated, CR-MASJ). The mean CR-MASJ scores improved from 2.12 at baseline to 0.68 (SD = 0.69) at *t* = 3 months and 1.27 (SD = 0.74) at *t* = 12 months

A more detailed and categorical analysis of the pairwise differences revealed that all patients (100%, 95% CI 91.4–100%) improved (either 1 or 2 points) from baseline *t* = 0 to post-treatment *t* = 3 on the CR-MASJ scale, and that 43.9% (95% CI 29.9–59.0%) of the patients improved as much as 2 points on the scale (Fig. 6). With respect to the change from post-treatment *t* = 3 to *t* = 12, there were no patients who relapsed 2 or more points on the scale. In total, 58.5% (95% CI 43.4–72.2%) of the patients relapsed 1 point from *t* = 3 to *t* = 12.

### Baseline by Treatment Interaction Effect

The observed range and standard deviation of baseline CR-MASJ signify considerable variation. It is therefore possible that for patients who start relatively high on CR-MASJ at baseline, the treatment may be more effective. This is known as a baseline by treatment interaction effect [22]. In the present study, the treatment effect is estimated by the proportion of patients who improved substantially (i.e., 2 points) on the CR-MASJ scale, and the baseline by treatment interaction effect is estimated here by the degree of association between baseline CR-MASJ level and the likelihood of a patient improving 2 points on the CR-MASJ scale. A logistic regression analysis showed that the association between baseline CR-MASJ level and the odds of improving 2 points on the CR-MASJ scale was statistically significant (Likelihood ratio test: Chi-square = 13.48, *p* < 0.001). The odds ratio (OR) of 5.82 (95% CI 2.11–21.73) suggests that an additional point on baseline CR-MASJ is associated with an increased odds of improving 2 points on the CR-MASJ scale by a factor of 6.

However, because injection volume is strongly associated with the baseline level CR-MASJ score, the former variable may be a confounder. To find out if confounding may occur here, the logistic regression was re-run with injection volume as a control variable. The results indicated that, statistically controlled for injection volume, the association between baseline CR-MASJ level and the odds of improving was now indeed less pronounced (OR = 3.39, 95% CI 1.03–14.21) but still statistically significant (Likelihood ratio test: Chi-square = 4.06, *p* = 0.044). Injection volume was, controlling for baseline CR-MASJ score, not a significant predictor in the logistic regression model (Likelihood ratio test Chi-square = 2.45, *p* = 0.118), with an OR of 1.31 (95% CI 0.94–1.96).

### Adverse Events

No adverse events other than injection site reactions were reported for any of the subjects at any given time.

## Discussion

Soft tissue fillers have gained impressive popularity in the past decades [1]. Facial aging is a complex, three-dimensional process that takes place in all existing tissue layers and therefore requires a holistic approach [17, 23, 24]. Due to the combination of the volume loss in the midface and laxity of the different tissue layers, the tissue moves to a caudal direction, resulting in a relative tissue surplus in the lower face over time. Hyaluronic acid-based fillers (HAs) are the most commonly used, followed by calcium hydroxylapatite (CaHA) [1]. The HAs modus operandi is mainly through direct volume replacement. CaHA is classified as a biostimulatory filler, since its effect is mediated by neocollagenesis [2], making it suitable for volumization, tissue-lifting and for skin-tightening procedures by hyper-diluting the product with lidocaine or saline [4, 5]. Compared to HAs, CaHA was shown to result in a more active, physiologic remodeling of the extracellular matrix by stimulating a two-step process where collagen type I gradually replaced type III [25].

In our previous study, we evaluated the safety of premixing CaHA and a CPM-HA (CaHA:CPM-HA) before injection, by reviewing the current literature on this topic and a retrospective chart review of 134 patients injected with a premixed combination of CaHA:CPM-HA (accepted data). Patient records were evaluated for adverse events at 1-3 month, 5-7 month and >12 month post-treatment follow-up visits. Only two adverse events were reported which comprised slight cases of overcorrection at 1–3 months. The combined evaluation of the published literature and retrospective examination of our cohort raised no concerns about the use of CaHA:CPM-HA for the treatment of facial aesthetic indications.

In this study, the authors investigate the improvement in facial contouring and duration of the premixed CaHA:CPM-HA combination. Numerous grading systems exist to assess and score skin quality, the amount and depth of lines and wrinkles, and volume loss. However, we found no grading system that directly scores the lifting effect of minimally invasive or surgical procedures of the face. Volume loss, tissue laxity and soft tissue ptosis are interrelated and all the result of facial aging. Therefore, the lifting effect of a treatment in the upper- or midface is visible by reduced soft tissue ptosis and thus aesthetic improvement of the lower face [16, 26]. Another way of measuring the lifting effect of filler treatments has been performed by 3D imaging with Vectra software (Canfield Scientific Inc., Parsippany, USA) [27]. However, since the data of this study were obtained retrospectively, we could not apply this measuring method for our study.

Restoration or lifting of the jawline contour knows several approaches, using cannulas or needles. Depending on the location, the product that is used, or the practitioner, injection can be performed by serial puncture (needle), tunneling, and linear threading. In a study from 2017, Baspeyras et al. published their results on jawline contour restoration using CaHA [16]. For the lower jawline, the authors used insertions at the mandibular angle and prejowl sulcus. For the upper jawline, the used insertions were at the posterior cheek and cheek bone (both in the midface). CaHA was injected using a blunt canula and fanning technique. Jawline contour improvement was assessed using the Merz Aesthetics Scale^®^ for jawline, which improved from 2.42 at baseline to 1.02 at day 30/60 (*p*≤0.0001), 1.11 at day 180 (*p* ≤ 0.0001), and 1.45 at day 360 (*p*=0.0015). The mean total volume of CaHA injected ranged from a mean of 3.90 mL in subjects with mild sagging (score 1) at baseline to 6.68 mL in subjects with mild-to-moderate sagging (score 1.5) [16]. Bertossi et al. performed a redefinition of the jawline in 30 subjects, using a high G’ and high cohesivity dermal filler (25mg/mL HA; Volux^®^, Vycross^TM^ range, Allergan, Dublin, Ireland) [28]. Injections were performed according to the MD Codes for the jawline and chin, consisting per side of the face of 5 points on the chin, 3 points at the prejowl sulcus, 2 points at the corner of the mouth, and 3 point around the mandibular angle [29]. A mean quantity of 4.0 ± 0.8mL of product was injected. Outcome was rated using the 5-point GAIS patient satisfaction score. 29 out of 30 patients (96.7%) rated their appearance after the treatment as ‘much improved’ or ‘very much improved’ [28].

Moradi et al. published the first study premixing CaHA and a high G’ HA filler (Juvéderm^®^ Voluma XC, Allergan, Dublin, Ireland; or Perlane^®^, Galderma, Lausanne, Switzerland) for chin and jawline augmentation [3]. The authors introduce new anatomical zones and nomenclature and divide the jawline aesthetic unit into three separate anatomical zones: masseteric, buccal, and mental. A combination of 29- to 27-gauge needles, or a 27-gauge blunt tip cannula were used, to better define the angle of the mandible and re-volumize the pre- and postjowl hollows. As a result of their approach, the jawline appeared visibly straighter. Their article was descriptive and did not include treatment of a patient cohort and assessment of treatment results [3]. Chang et al. also describe the use of premixed CaHA and HA (CPM-HA B) for facial rejuvenation in a cohort of 25 patients that scored 1 or 2 on the Merz Aesthetics Scale^®^ for nasolabial fold (NLF) and jawline [11]. The NLF and jawline were injected with the premixed CaHA:CPM-HA using a 27-gauge blunt-tipped cannula in a fanning pattern. A visual analog scale (VAS) and the 5-point global satisfaction scale (GSS) were used for objective and subjective treatment assessments. In a subset of patients, 0.1mL of the mixture and 0.1mL of only calcium hydroxylapatite filler were injected into the right and left postauricular areas, and biopsies were taken at *t* = 6 months for histological analysis. For the nasolabial folds, the mean VAS score was 7.0 of 10 at 1 month and 5.8 at 9 months. For the jawlines, the mean VAS score was 7.4 at 1 month and 6.4 at 9 months. For the nasolabial folds, the mean GSS score was 4.8 of 5 at 1 month, and 3.4 at 9 months. For the jawlines, the mean GSS score was 4.7 at 1 month and 3.2 at 9 months. The mean VAS and GSS scores decreased significantly over time (*P* < 0.05) but the mean scores of VAS and GSS were above ‘‘fair’’ at all follow-up points. The histological specimens at 6 months after the injection showed newly formed, irregular, thick collagen bundles in the dermis in both groups.

These discussed studies show that restoration of the jawline contour is an effective and well-appreciated rejuvenation procedure which can be achieved by different injection techniques and with different products. Chang *et al.* chose to premix CaHA and a CPM-HA to compensate for the rapid absorption of the methylcellulose carrier of Radiesse^®^ which is observed in some cases. In a study by Godin et al., they showed that combining Radiesse^®^ and Restylane^®^ (Galderma, Lausanne, Switzerland) provided higher satisfaction than using Radiesse^®^ alone.^(9)^ In our study, we chose to premix CaHA and CPM-HA in order to take advantage of the complementary mechanisms of action of both fillers. In our previous study, we demonstrated that the use of this hybrid mix was safe in a cohort of 134 patients and a follow-up time of 1 year [13]. In this study, the obtained data show that 100% of the included subjects had experienced a ≥1-point improvement in CR-MASJ score at *t* = 3 months (95% CI 1.28 to 1.60), versus 85% at *t* = 12 months (95% CI 0.74–0.97). In 43.9% (*n* = 18) of cases, a 2-point improvement was achieved at *t* = 3 months (95% CI 29.9–59.0%). This supports the volumizing and lifting effectivity of the hybrid mix CaHA:CPM-HA, by injecting cheeks and jawline as done in this study. The mean CR-MASJ score improved from 2.12 at baseline to 0.68 at *t* = 3 months (SD = 0.69, 95% CI 1.28–1.60) and 1.27 at *t* = 12 months (SD = 0.74, 95% CI 0.43–0.74). These data are comparable to the above-mentioned studies. The ratio between the score at baseline (CR-MASJ = 2.12) and at *t* = 3 months (CR-MASJ = 0.68) was 0.32 (0.68/2.12) in our study, and 0.42 (1.02/2.42) for the Baspeyras study [16]. Suggesting the improvement was superior in our cohort. Interestingly, the ratio between baseline and *t* = 12 months was 0.60 for both this study and Baspeyras et al. (1.27/2.12 vs. 1.45/2.42). This might underscore the result from Godin et al. suggesting that premixing an HA with CaHA yields higher satisfaction than CaHA alone, since one would expect the effect of the HA to last up to 1 year [9]. Though many HAs do not maintain their effect that long. One might also argue that at *t* = 12 months only the effect of CaHA remains, supporting one of the premixing goal of adding time to the treatment effect compared to treating with HA alone.

### Limitations

This study investigated facial treatment with a hybrid mix of CaHA and CPM-HA in a cohort of 41 patients. Although this cohort can be considered substantial, further research with more subjects would lead to more valuable results. Due to its retrospective character, the results were not evaluated by the patients themselves, and no 3D imaging and measurements could be applied afterwards. Blinding, randomization or adding a control group was logistically not possible. The treatments were performed with different premixing ratios. Although this creates a bias in the results and should be considered as a limitation, this approach was necessary for achieving the desired aesthetic results. Despite these limitations, we believe that the findings represent valuable information for clinical practice.

## Conclusion

The results of this study support the volumizing and lifting effectivity of the hybrid mix CaHA:CPM-HA, by injecting cheeks and jawline. In addition, the results suggest that premixing an HA with CaHA yields a higher satisfaction than injecting CaHA alone at *t* = 3 months. This advantage disappears at *t* = 12 months, which could correlate to the ended effect of the HA component of the hybrid CaHA:CPM-HA mix.
